# Mesalamine-Mediated Amelioration of Experimental Colitis in Piglets Involves Gut Microbiota Modulation and Intestinal Immune Cell Infiltration

**DOI:** 10.3389/fimmu.2022.883682

**Published:** 2022-07-08

**Authors:** Yonggang Huang, Miaomiao Wu, Hao Xiao, Hongnan Liu, Guan Yang

**Affiliations:** ^1^ Animal Nutritional Genome and Germplasm Innovation Research Center, College of Animal Science and Technology, Hunan Agricultural University, Changsha, China; ^2^ State Key Laboratory of Livestock and Poultry Breeding, Institute of Animal Science, Guangdong Academy of Agricultural Sciences, Guangzhou, China; ^3^ Key Laboratory of Agro-ecological Processes in Subtropical Region, Institute of Subtropical Agriculture, Hunan Provincial Key Laboratory of Animal Nutritional Physiology and Metabolic Process, Chinese Academy of Sciences, Changsha, China; ^4^ Department of Infectious Diseases and Public Health, City University of Hong Kong, Kowloon, Hong Kong SAR, China

**Keywords:** colitis, gut microbiota, immune cells, mesalamine, piglet

## Abstract

Mesalamine (MES), also known as 5-aminosalicylic acid, is effective in treating mild to moderate ulcerative colitis (UC). The mechanisms of its actions are not fully elucidated. The aim of this study was to investigate the effects of MES treatment on intestinal microbiota and immune system in an dextran sulfate sodium (DSS)-induced UC model in postweaning piglets. Eighteen weaned piglets were assigned randomly to the following treatments: control group (CON, distilled water), DSS group (DSS, 3% DSS), and MES group (MES, 3% DSS + 2 g/day MES). Our results showed that MES treatment alleviates DSS-induced colitis in piglets, as evidenced by a reduced diarrhea index score and increased average daily gain (*P* < 0.05). This is accompanied by decreased diamine oxidase activity, D-lactate level (*P* < 0.05), and attenuated mucosal damage. MES treatment also decreased the abundance of *Methanogens* and reduced colon CD11b^+^ macrophage and CD3^+^ T-cell infiltrations in piglets with DSS-induced colitis (*P* < 0.05). Collectively, these data indicate that MES treatment-mediated colitis protection may involve microbiota and immune cell alterations.

## Introduction

Intestinal barrier damage, diarrhea, and weight loss are common features of inflammatory bowel diseases (IBD), encompassing ulcerative colitis (UC), and Crohn’s disease (CD) (1). These diseases substantially reduce the quality of life and lack specific treatments (2, 3). IBD pathogenesis involves genetic and environmental factors, such as alterations in microbiota (4). Patients with IBD exhibit an altered microbiome profile compared to healthy controls, and manipulation of microbiota has potential for IBD therapy (5–7). Since the gut microbiota plays an important role in regulating host immunity, dysregulation of the gut microbiota can lead to a host immune disorder, which can trigger chronic inflammatory diseases such as IBD (8). In recent years, the relationship between gut microbiota and host immunity has become a popular research direction in the treatment of IBD (9).

Indeed, the intestinal microbiota interacts with the host mucosal immune system to maintain immune homeostasis. Patients with IBD lack immune tolerance to commensal microbiota, leading to exacerbation in immune reaction against the intestinal flora (10). The microbiota–host immune system interaction is further evidenced by studies using germ-free mice, which exhibited decreased numbers of colonic CD4^+^ T cells and were protected in several experimental IBD models (11). Specific gut bacterial species, such as *Clostridia* and *Bacteroides*, are critical in mucosal immunity through regulating Treg-cell differentiation and function (12).

Mesalamine (MES) is a first-line drug for the treatment and relief of moderately mild UC (13). Studies have found that MES is effective in treating UC *via* inhibiting various signaling pathways, including Wnt/β-catenin, PPAR-γ, NF-κB, MAPK, PI3K/Akt, and aryl hydrocarbon receptor (AhR) (14–17). Gut microbes may have a direct impact on the efficacy of IBD drugs through biotransformation (18). However, the effects of MES on the gut microbiota when treated with UC are poorly studied. In this study, we evaluated the effects of MES treatment on intestinal immune cells and microbiota in DSS-induced colitis in piglets, which are similar to humans in anatomical size, physiology, and immunology. We hypothesized that MES contributes to UC alleviation by restoring intestinal flora, reducing intestinal intraepithelial lymphocyte (IEL) migration toward the apical epithelium, and affecting cytokine–cytokine receptor signaling.

## Materials and Methods

### Animals and Experimental Design

**Figure 1:**
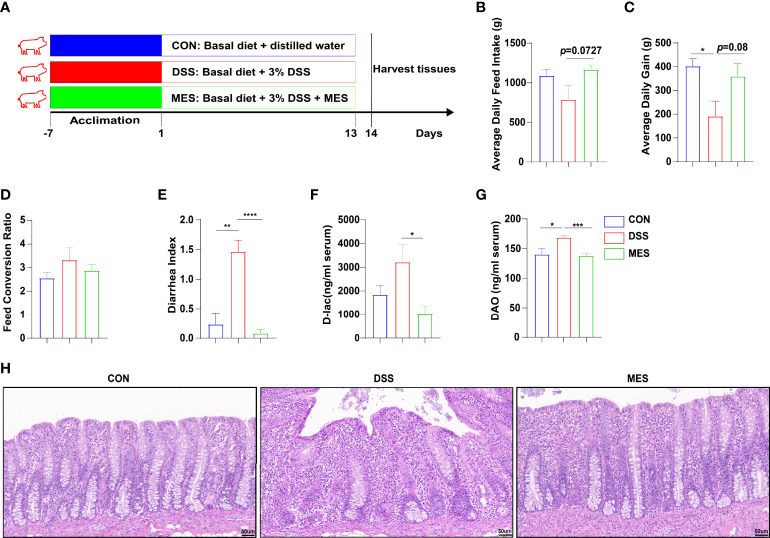
MES alleviates DSS-induced colitis in piglets. **(A)** Schematic diagram of the colitis model of weaned piglets used in this study; **(B)** average daily feed intake, **(C)** average daily gain, and **(D)** feed conversion rate during DSS treatment; **(E)** diarrhea index; **(F)** serum levels of D-lactic acid and **(G)** serum diamine oxidase; **(H)** H&E-stained colon sections. Scale bar = 50 µm. Data were presented as mean ± SEM (n = 6 per group). Statistical significance was determined using one-way ANOVA, followed by Tukey test. *P < 0.05, **P < 0.01, ***P < 0.001, ****P < 0.0001.

All animal procedures were performed with an approved protocol by the Animal Ethics Committee of Hunan Agricultural University. The experimental design is shown in **Figure 1A**. After a 7-day adaptation period, 18 healthy piglets (Duroc × Landrace × Large Yorkshire) weaned at 28 days of age (average body weight: 8.99 ± 0.4 kg) were randomly assigned to three groups (six pigs/group). The treatment groups are as follows: (1) CON group: daily oral administration of 12 ml distilled water for 13 days; (2) DSS group: daily oral administration of 12 ml 3% (w/v) DSS (MW = 40 kDa, Aladdin, Shanghai, China) for 13 days; (3) MES group: daily oral administration of 12 ml 3% DSS and 2 g of MES (Jiamusi Luling Pharmaceutical Co., Ltd., China) for 13 days. The piglets were housed individually in an environmentally controlled nursery with hard plastic-slatted flooring and had free access to standard diet and drinking water. Average daily gain (ADG), average daily feed intake (ADFI), feed conversion ratio (FCR), and diarrhea index were calculated. The diarrhea index was calculated by adding the diarrhea scores of each piglet during DSS modeling and divided by the number of piglets in each group. The diarrhea index was scored according to the viscosity of feces, and the scoring standard was as follows: 0 = normal; 1 = soft feces; 2 = mild diarrhea; 3 = severe diarrhea (19). All piglets were sacrificed on day 14, and serum was obtained by centrifugation (4,000 × rpm for 10 min at 4°C) and then stored at − 80°C. Colon and jejunum tissues were flushed with PBS and then fixed in 10% formalin for subsequent histology analysis and immunofluorescence assay (IFA). Liquid nitrogen quick-frozen colon samples were used for RNA sequencing analysis, and colon digesta was used for 16S rRNA sequencing by Beijing Novogene Bioinformatics Technology Co., Ltd. (China).

### Determination of Intestinal Permeability and Inflammatory Factors

Alanine transferase (ALT) and globulin (GLB) contents in serum were detected by an automatic biochemical analyzer (Kehua Biology, Shanghai, China). Serum levels of diamine oxidase (DAO), D-lactate (D-lac), myeloperoxidase (MPO), interleukin 22 (IL-22), IL-12p70, and IL-1β were determined by enzyme-linked immunosorbent assay (ELISA) kits (Jiangsu Meimian Industrial Co., Ltd., China).

### Histological Analysis and Immunofluorescence Staining

Formalin-fixed colon and jejunum tissues were paraffin-embedded, and 5-µm-thick sections were either stained with hematoxylin and eosin (H&E) or processed for IFA. The staining quality of colon sections was observed under a normal white light microscope. Digital scanning imaging was performed using an orthopedic light microscope (Nikon, Tokyo, Japan). The images were evaluated using the CaseViewer 2.4 software (3DHISTECH, Budapest, Hungary). The extent of inflammatory infiltration, histopathological changes in crypt structure, ulceration, crypt loss in the colon, and villus height and crypt depth in jejunum were measured as previously described (20, 21). IFA sections were set on poly-L-lysine-coated glass slides, deparaffinized in xylene, and rehydrated through graded concentrations of ethanol bath to distilled water, followed by heat-induced epitope retrieval. Slides were blocked with 5% bovine serum albumin (BSA) (Sigma, St. Louis, MO, USA) and incubated overnight at 4°C with primary antibody to CD11b (1:50, Abcam, Cambridge, MA, USA), CD3 (1:50, Proteintech Group, Chicago, IL, USA), CD79a (1:50, Thermo Fisher Scientific, Waltham, MA, USA), or aquaporin 3 (AQP3) (1:50, Abcam, USA) and washed three times for 5 min each in PBS (PH 7.4) and then incubated with 50 ml secondary antibody, CoraLite594-conjugated goat anti-rabbit IgG H&L (Abcam, USA). DAPI working solution was stained for 10–20 min at 37°C and washed three times with PBS for 5 min each. Staining was examined using a fluorescence microscope (Motic, China) after glycerin sealing. Quantitative analysis of the expression of relevant proteins by software Image-Pro Plus 6 (Media Cybernetics, Rockville, MD, USA).

### Reverse Transcription-Quantitative PCR

Total RNA of colon tissues was extracted using TRIzol reagents (Invitrogen, Carlsbad, CA, USA) according to the manufacturer’s instructions. The quality and purity of RNA were assessed by absorbance at 260 nm and 280 nm by using a Nanodrop spectrophotometer (Thermo Fisher Scientific, USA). Reverse transcription of 10 µl RNA was conducted to synthesize cDNA using 5 × PrimeScript Buffer 2 and PrimeScript reverse transcriptase Enzyme Mix 1 (TaKaRa Biotechnology, [Dalian] Co., Ltd., China). The primers used to amplify genes are shown in **Supplementary Table 1**. Quantitative PCR was performed on a LightCycler^®^ 480 Real-Time PCR system (Roche, Basel, Switzerland). Average Ct values from triplicate analyses were normalized from average Ct values of β-actin, and data were expressed as relative to those in the CON group.

### RNA Sequencing

RNA extraction, quality verification, library preparation, sequencing, and transcriptomic analysis were performed by Beijing Novogene Bioinformatics Technology Co., Ltd. (China). Briefly, cDNA was synthesized from purified mRNA and used to amplify RNA. The integrity of PCR products was assessed using the RNA Nano 6000 Assay Kit of the Bioanalyzer 2100 system (Agilent Technologies, Santa Clara, CA, USA). Clean data were acquired by filtering reads with adapter, poly-N, and Illumina low-quality reads from raw data and mapped to the reference genome using Hisat2 (version 2.0.5). Quantification of gene expression level was performed using featureCounts v1.5.0-p3. Differential expression analysis was conducted using DESeq2, and adjusted *P* value < 0.05 was considered as the threshold for significantly differential expression. Gene Ontology (GO) enrichment analysis of differentially expressed genes was implemented, and we used clusterProfiler R package to test the statistical enrichment of differential expression genes in Kyoto Encyclopedia of Genes and Genomes (KEGG) pathways by the cluster Profiler R package.

### Microbiota Analysis Based on 16S rRNA High-Throughput Sequencing

Total microbial genomic DNA from samples was extracted using the QIAamp DNA Isolation Kit (Qiagen, Hilden, Germany). DNA concentration, purity, and integrity were examined using agarose gel electrophoresis. Based on these parameters, qualified samples were then selected and diluted to 1 ng/ml to conduct the subsequent sequencing. DNA samples were used as a template to amplify the V4-hypervariable region of the bacterial 16S rRNA gene with barcoded primers (515 forward primer, 5′-GTGCCAGCMGCCGCGGTAA-3′, 806 reverse primer, 5′-GGACTACHVGGGTWTCTAAT-3′) and Phusion^®^ High-Fidelity PCR Master Mix with GC Buffer (New England Biolabs, Ipswich, MA, USA). PCR amplification products were subjected to electrophoresis on 2% agarose gel and purified with Qiagen Gel Extraction Kit (Qiagen, Germany). Sequencing libraries were constructed using TruSeq^®^ DNA PCR-Free Sample Preparation Kit (Illumina, San Diego, CA, USA), and library quality was evaluated on Qubit 2.0 Fluorometer (Thermo Fisher Scientific, USA). Purified PCR products were then sequenced using the Illumina NovaSeq platform (Thermo Fisher Scientific, USA) with 250-bp paired-end reads. Raw microbiota data were analyzed utilizing the QIIME platform (version 1.9.1). Raw tags were filtered according to the QIIME quality-controlled process, and effective tags were acquired after chimera sequences were detected and removed based on the Silva database. Operational taxonomic units (OTUs) were selected using Uparse software (version 7.0.1001) with a clustering threshold of 97%. Alpha and beta diversities and environmental factor correlation (Spearman analysis) were calculated with QIIME (Version 1.7.0) and displayed with R software (Version 2.15.3). Principal coordinate analysis (PCoA) plots were displayed by WGCNA package, stat packages, and ggplot2 package in R software (version 2.15.3). Illumina MiSeq sequencing, processing of sequencing data, and bioinformatics analysis were performed by Beijing Novogene Bioinformatics Technology Co., Ltd. (China).

### Environmental Factor Correlation Analysis

According to the relative abundance at the genus level, gut microbiome abundance data for the top 35 taxa (genus level) were used for correlation analysis with immune-related differently expressed genes (DEGs). Spearman correlation was used for this analysis, as it performs better with normalized counts (gene expression) and compositional data (microbiome relative abundance).

### Statistical Analysis

GraphPad Prism 8 software (USA) and SPSS 22.0 Statistical Software (USA) were adopted for statistical analysis. Data were presented as mean ± SEM. One-way ANOVA was used to compare the difference among groups, followed by Tukey’s multiple-comparison tests. *P* < 0.05 was considered statistically significant.

## Results

### MES Treatment Improves Intestinal Permeability in DSS-Induced Colitis in Piglets

To study the effects of MES treatment on colitis, experimental colitis was induced in piglets by administering DSS for 13 days continuously (22, 23), and 2 g MES was given by oral administration (**Figure 1A**). MES treatment alleviated DSS-induced colitis in piglets, as evidenced by increased ADG and reduced diarrhea index (*P* < 0.05) (**Figures 1B–E**). MES treatment also increased ADFI (*P* < 0.05) but did not affect FCR (**Figure 1D**). D-Lac is released by gut microflora and DAO is synthesized only in intestinal epithelia, and increased blood levels of these parameters reflect mucosal permeability and abnormal barrier function (24). We found that MES treatment decreases serum concentrations of D-lac and DAO in DSS-treated piglets (*P* < 0.05) (**Figures 1F, G**). Histological analysis further demonstrated obvious attenuation of epithelial erosion and crypt loss in response to MES treatment (**Figure 1H**). In accordance with other published reports (25), our study also showed that MES treatment decreased the serum concentrations of ALT and GLB in colitis pigs (*P* < 0.05) (**Figures S1A, B**). In addition, MES treatment increased the ratio of jejunum villus height to crypt depth induced by DSS (*P* < 0.05) (**Figures S1C–F**). These results collectively indicate that MES treatment ameliorates clinical symptoms, gut barrier injury, liver damage, and intestinal permeability induced by DSS.

### MES Treatment Alters Immune-Related Gene Expression

**Figure 2:**
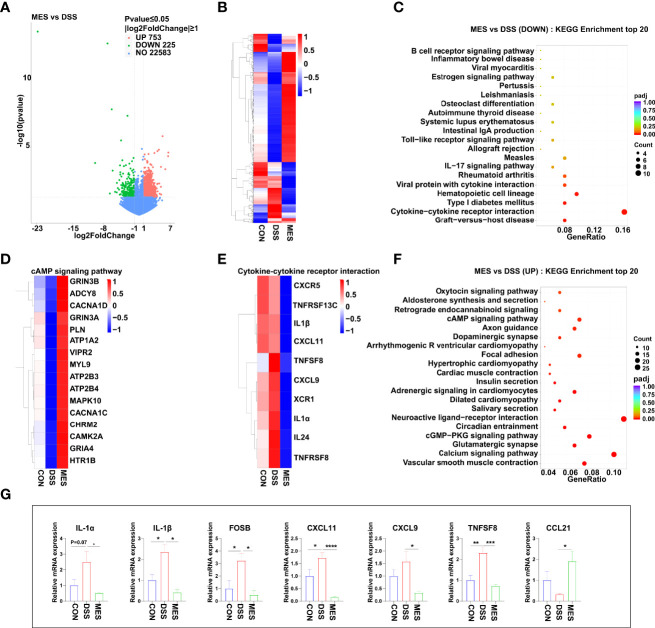
MES suppresses immune responses in piglets with DSS-induced colitis. **(A)** Volcano plot of DEGs between DSS and MES groups. The X-axis represents the fold change in the expression of DEGs, and the Y-axis represents the statistical significance of the fold change. Each point represents a DEG. **(B)** Cluster heatmap of DEGs; top 20 KEGG annotation of **(C)** downregulated and **(F)** upregulated DEGs. The numbers of DEGs in each pathway are counted, and the KEGG gene ratio and adjusted *P*-values are acquired by hypergeometric distribution test. Heatmap of DEGs enriched in cAMP signaling pathway-related KEGG pathways **(D)**, cytokine–cytokine receptor interaction **(E, G)** Relative mRNA abundances of IL-1α, IL-1β, CXCL11, CXCL9, CCL21, FOSB, and TNFSF8 validated by RT-qPCR. Values were means ± SEM (n = 6 per group). *P < 0.05, **P < 0.01.

To obtain a better understanding of the potential effects of MES treatment on piglet colitis, we assessed the global transcriptomic profiles of colon tissues from CON, DSS, and MES groups. RNA sequencing analysis revealed significant changes in transcript levels. MES treatment markedly induced (n = 753) or reduced (n = 225) the gene expression compared to the DSS group (**Figure 2A**). The hierarchical cluster heatmap showed that the expression profiles of DEGs in the MES group are distinctly different from the DSS group (**Figure 2B**). Interestingly, KEGG pathway analysis showed that MES treatment affects immune response-related pathways, especially down-regulates the cytokine–cytokine receptor interaction and hematopoietic cell lineage (*P* < 0.05) (**Figures 2C, E**), and up-regulates the cAMP signaling pathway, cGMP-PKG signaling pathway, neuroactive ligand–receptor interaction, and calcium signaling pathway (*P* < 0.05) (**Figures 2D, F**). We next validated these cytokine- and cytokine receptor-associated gene expressions by RT-qPCR. Consistently, our results showed that compared with the DSS group, MES treatment reduces *IL-1α*, *IL-1β*, *CXCL11*, *CXCL9*, *FOSB*, and *TNFSF8* mRNA expressions and enhances *CCL21* expression (*P* < 0.05) (**Figure 2G**). Taken together, these results suggest that the protective actions of MES for treating UC are associated with immune-related gene expression reprogramming.

### MES Treatment Suppresses DSS-Induced Inflammation

**Figure 3:**
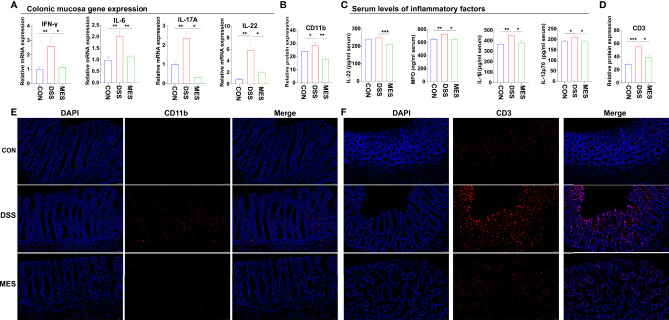
MES reduces the recruitment of immune cells in piglets with DSS-induced colitis. **(A)** Colonic mucosa mRNA levels of IL-6, IL-17A, IL-22, and IFN-γ; **(B, D)** intestinal frequencies of CD11b^+^ macrophages and CD3^+^ T cells; **(C)** serum levels of MPO, IL-1β, IL-22, and IL-12 p70; **(E, F)** representative images of intestinal infiltrations of CD11b^+^ myeloid cells and CD3^+^ T cells. Scale bar = 50 µm. Data were presented as mean ± SEM (n = 6 per group). Statistical significance was determined using one-way ANOVA, followed by Tukey test. *P < 0.05, **P < 0.01, ***P < 0.001.

To further assess the impact of MES treatment on the systemic and intestinal inflammatory responses caused by DSS administration, we determined the levels of some pro-inflammatory cytokines and MPO in colon mucosa and serum. We found that MES treatment reduces proinflammatory factor expression at the mRNA (*IFN-γ*, *IL-6*, *IL-17A*, and *IL-22*) and protein (IL-1β, IL-22, and IL-12p70) levels (*P* < 0.05) (**Figures 3A, C**). MES treatment also decreased the MPO concentration in the colon mucosa induced by DSS (*P* < 0.05) (**Figure 3C**). Because immune cells play a critical role in IBD, we next performed IFA using antibodies against CD11b (**Figure 3E**), CD3 (**Figure 3F**), AQP3 (**Figure S2A**), and CD79a (**Figure S2B**). We found that MES treatment attenuates infiltrations of CD11b^+^ macrophages and CD3^+^ T cells induced by DSS (*P* < 0.05) but has no effects on CD79a^+^ B cells or AQP3^+^ cells (**Figures 3B, D**). The above results indicate that MES treatment suppressed DSS-induced inflammation.

### MES Treatment Alters Gut Microbiota Composition in DSS-Induced Piglet Colitis

**Figure 4:**
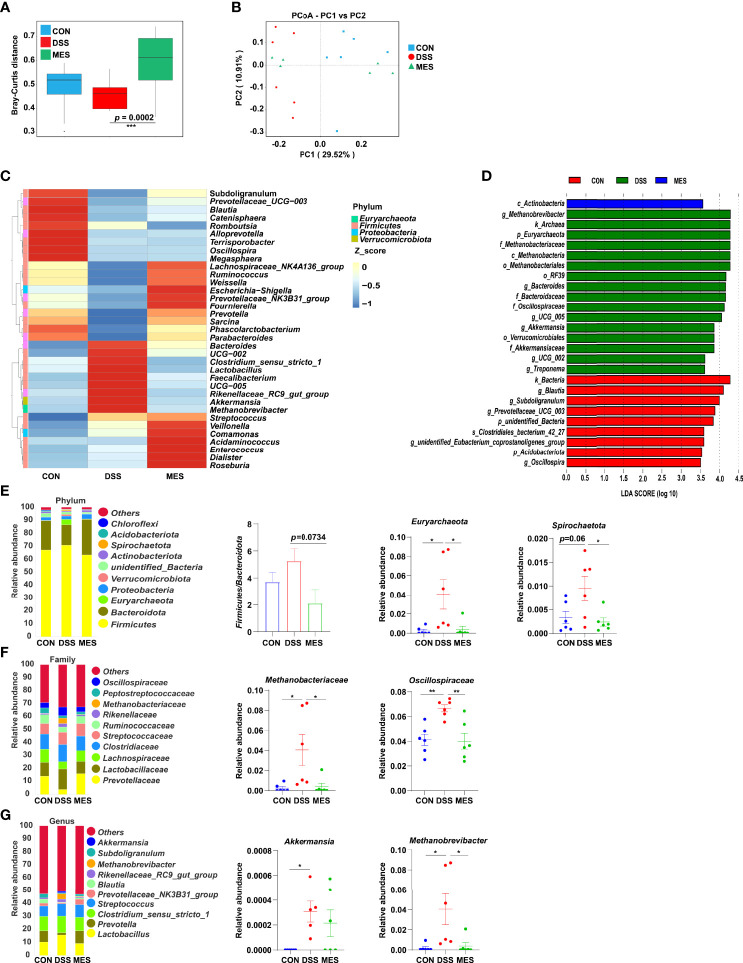
MES modulates gut microbiota in piglets with DSS-induced colitis. **(A)** Rank abundance curves of beta diversity in the CON, DSS, and MES groups; **(B)** PCoA plots upon MES therapy assessed by PERMANOVA; **(C)** heatmaps of species abundance at the genus level; **(D)** LEFSe analysis revealed the dominant bacteria in the CON, DSS, and MES groups; relative abundance of top 10 **(E)** phyla, **(F)** families, **(G)** genera in the CON, DSS, and MES groups. *P < 0.05, **P < 0.01, ***P < 0.001.

UC is typically associated with gut microbiota dysbiosis (26). Next, we investigated the impact of MES treatment on gut microbiota composition in DSS-induced colitis *via* 16S rRNA gene sequencing. Alpha diversity indexes (chao1, ace) were not impacted by MES treatment (**Figures S3A, B**), and beta diversity was markedly different between DSS and MES groups (*P* < 0.05) (**Figure 4A**). PCoA showed a separation in the gut microbiota structure between DSS and CON groups (R^2^ = 0.268, *p* = 0.005) (**Figure 4B**). Together, these data indicate that the gut microbiota structure in pigs with colitis was impacted by MES treatment. Linear discriminant analysis (LDA) combined with effect size measurement (LEfSe) was used to determine the key microbiota taxa. *Actinobacteria* were enriched in the MES group, while *Methanobrevibacter*, *Archaea*, and *Euryarchaeota* were enriched in the DSS group (**Figure 4D**). At the phylum level, MES treatment decreased the relative abundance of *Euryarchaeota* and *Spirochaetota* and tended to decrease the ratio of *Firmicutes* to *Bacteroidota* compared with the DSS group (*P* < 0.05) (**Figure 4E**). At the family level, MES treatment reduced the relative abundance of *Methanobacteriaceae* and *Oscillospiraceae* induced by DSS (*P* < 0.05) (**Figure 4F**). At the genus level, MES treatment reduced *Methanobrevibacter* abundance induced by DSS (*P* < 0.05) (**Figure 4G**). Specifically, we found that MES treatment significantly suppresses the abundance of *Methanobrevibacter* (belonging to the *Methanobacteriaceae* family), a common signature of the gut microbiota dysbiosis (27), induced by DSS (**Figure 4G**). Collectively, these results suggest that MES treatment protects against DSS-induced colitis in piglets, which could be due to its recovery function of gut microbiota dysbiosis.

### DSS Administration-Induced Proinflammatory Factors Are Correlated With *Methanobrevibacter*

**Figure 5:**
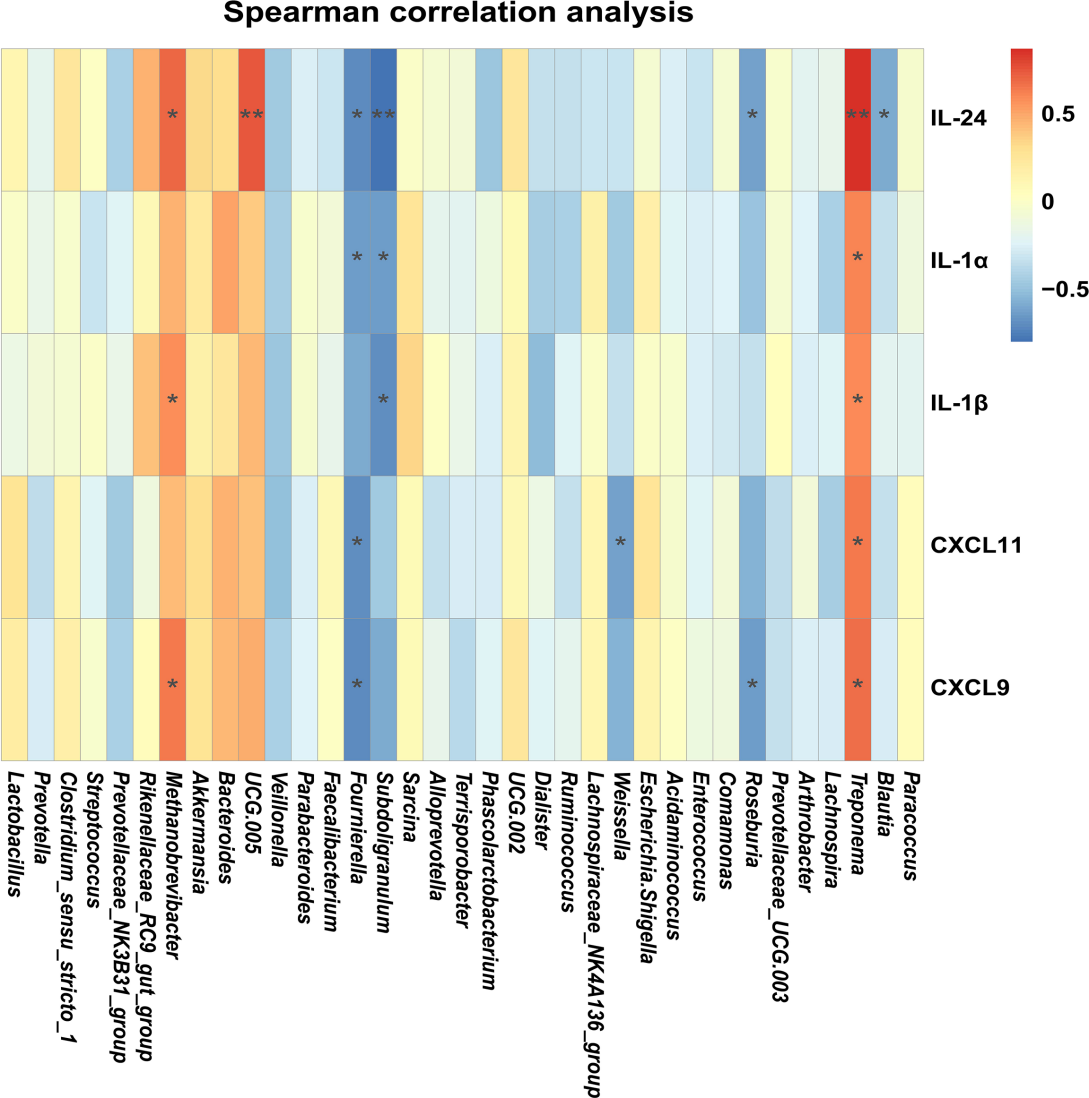
MES-mediated mitigative effects in piglets with DSS-induced colitis are correlated with gut microbiota alteration and colonic inflammation: heatmap of the Spearman correlation analysis between the gut microbiota and colonic proinflammatory factors.

In order to illustrate the relationships between proinflammatory factors and the genus-level bacteria driven by MES treatment, we performed Spearman correlation analysis. We identified significant interactions between some proinflammatory factors (*IL-24*, *IL-1β*, and *CXCL9*) and *Methanobrevibacter* (*P* < 0.05) (**Figure 5**). Expressions of these cytokines and chemoattractants were enhanced in the colon of colitis piglets and were inhibited by MES treatment. Thus, our data suggest that MES treatment-mediated protective effects on colitis might be correlated with reduced *Methanobrevibacter* enrichment.

## Discussion

MES plays a well-established role in the management of IBD, but its action on gut microbiota and host immunity is not fully understood (28). In this study, we found that MES treatment modulates *Methanobrevibacter*, reprograms colon gene expression profiles associated with cytokine–cytokine receptor interaction, and alters frequencies of intestinal CD11b^+^ myeloid cells and CD3^+^ T cells in DSS-induced colitis in piglets.

To explore how MES treatment attenuated the colitis, a mild-to-moderate colitis piglet model was induced by oral DSS administration and treated by oral MES. Akin to previous research (17), our data showed that therapeutic application of oral MES treatment ameliorates DSS-induced colitis. Considering that MES works on the luminal side of inflamed colonic mucosa, we propose that the colitis-protective effects of MES treatment were due to its impact on alterations of gut microbiota profile and host cell signaling.

Gut microbiota dysbiosis is implicated in the pathogenesis of UC (29). Results of the present study showed that MES can significantly increase beta diversity without altering alpha diversity. The structure of the microbial community, but not community richness, was impacted in piglets with colitis following MES treatment, indicating that MES treatment prevented UC probably by selectively altering microbiota composition rather than by sterilizing the gut. UC patients had increased relative abundance of *Bacteroidetes* (30, 31). In the current study, we observed that MES treatment restores the DSS-mediated increment of the *Firmicutes*/*Bacteroidetes* ratio and this reduction was attributed to the enrichment of *Bacteroidetes*. Colitis piglets also displayed a markedly increased abundance of the *Methanobrevibacter* genus and *Akkermansia* genus, *Methanobacteria* family, and the archaeal phylum *Euryarchaeota*, which were highly affected by MES treatment. Increased *Methanobacteria* and *Akkermansia* were negatively correlated with some anti-inflammatory cytokines, such as TNFp1 (32). However, how MES treatment reduces the *Methanobacteria* level is still unknown. It is highly possible that *Methanobacteria* genome sequences may also contain the polyphosphate kinase homologies, the accumulation of which sensitizes bacteria towards oxidative stress and is a target of MES (33).

Although the etiology of IBD remains unclear, it is thought to be caused by an intricate interaction between microbiota and immune cells leading to inflammation and ulceration (34). Many IBD risk loci are proved to be found in regions of genes encoding cytokines or their downstream signaling mediators (35–37). Intestinal cytokines play a key role in promoting or inhibiting IBD intestinal inflammation, and many cytokine-targeted therapies are being used or are being tested clinically (38). Thus, it is likely that reciprocal interactions between microbiota and immune cells are exerted partly *via* intermediary factors (such as cytokines). As expected, we observed a marked difference between DSS and MES groups in IL-17 signaling and cytokine–cytokine receptor interaction pathways. Interestingly, a previous study revealed that IFN-γ, IL-1β, IL-6, IL-17, and IL-22 production exhibits specific associations with gut microbial dysbiosis in IBD (39). Our results showed that MES treatment also reduced gene/protein expressions including IFN-γ and Th-17-related cytokines induced in colitis piglets. Moreover, correlation analyses indicated that *Methanobrevibacter* are positively associated with changes in levels of *IL-1β*, *IL-24*, and *CXCL9* in the MES group. These results indicate the important role of microbiota and cytokine in MES alleviation on piglets with colitis.

Evidence also suggests a causal association between intestinal inflammation and altered monocyte-macrophage polarization. These macrophages are actively involved in impaired gut bacterial clearance (40, 41) and excessive proinflammatory cytokine secretion (42–45) in IBD patients. Meanwhile, recent single-cell analyses of IBD tissues revealed a potential link between inflammation and altered distribution of intestinal intraepithelial T cells (46). Here, our data showed that MES treatment reduces frequencies of intestinal myeloid cells and CD3^+^ T cells in a current DSS-induced colitis piglet model. This is partly evidenced by an elevated expression of T-cell chemoattractants such as *CXCL9*, *CXCL11*, and *CCL21* in colonic epithelial cells in piglets with DSS-induced colitis. Collectively, our results revealed that MES reduces intestinal myeloid cells and CD3^+^ T-cell recruitments, which could be a possible therapeutic target.

## Conclusion

In conclusion, our data showed that MES treatment-attenuated DSS-induced piglet colitis is related to the regulation of microbial immunity, inflammatory cytokines, and intestinal immune cells. Interestingly, our results also identified that methanogenic Archaea, *Methanobacteria*, could be a potential therapeutic target for treating IBD. The anti-inflammatory mechanisms of MES in DSS-induced colitis are related to gut enrichment of methanogenic Archaea, cytokine production, and intestinal immune cell recruitments. Thus, our findings have important implications for the development of IBD therapies and may provide novel insights into the efficacy of drugs that target Archaea to improve preventive strategies for IBD and other inflammatory disorders.

## Data Availability Statement

The original contributions presented in the study are publicly available. This data can be found here: http://www.ncbi.nlm.nih.gov/sra/PRJNA812765 and https://www.ncbi.nlm.nih.gov/sra/PRJNA815676.

## Ethics Statement

The animal study was reviewed and approved by Animal Ethics Committee of Hunan Agricultural University. Written informed consent was obtained from the owners for the participation of their animals in this study.

## Author Contributions

All authors contributed to the writing, review, and editing of the manuscript. MW and GY designed the experiments. YH and HX conducted the experiments. YH collected the samples and performed the analysis of samples. HL and HX analyzed the data. MW and GY wrote the manuscript. MW and GY contributed to researching the content for the article. MW and GY made substantial contributions to the discussion of content. All authors read and approved the final manuscript.

## Funding

This work was supported by the National Natural Science Foundation of China (Project number: 32002188), the new research initiatives at City University of Hong Kong (Project number: 9610541), National Natural Science Foundation of China (Project number: 32072741), and Youth Innovation Promotion Association (CAS, 2019356).

## Conflict of Interest

The authors declare that the research was conducted in the absence of any commercial or financial relationships that could be construed as a potential conflict of interest.

## Publisher’s Note

All claims expressed in this article are solely those of the authors and do not necessarily represent those of their affiliated organizations, or those of the publisher, the editors and the reviewers. Any product that may be evaluated in this article, or claim that may be made by its manufacturer, is not guaranteed or endorsed by the publisher.

## Abbreviations

ADFI, average daily feed intake; ADG, average daily gain; AhR, aryl hydrocarbon receptor; ALT, alanine transferase; AQP3, aquaporin 3; CD, Crohn’s diseases; CSF1, colony-stimulating factor 1; DAO, diamine oxidase; DEGs, differently expressed genes; D-lac, D-lactate; DSS, dextran sulfate sodium; ELISA, enzyme-linked immunosorbent assay; FCR, feed conversion ratio; GLB, globulin; GO, Gene Ontology; H&E, hematoxylin and eosin; IBD, inflammatory bowel diseases; IELs, intraepithelial lymphocytes; IFA, immunofluorescence assay; IFN-γ, interferon-gamma; KEGG, Kyoto Encyclopedia of Genes and Genomes; LDA, linear discriminant analysis; LEfSe, linear discriminant analysis effect size; LPS, lipopolysaccharide; MES, mesalamine; MPO, myeloperoxidase; MS, multiple sclerosis; PCoA, principal coordinate analysis; RT-qPCR, reverse transcription quantitative polymerase chain reaction; Tregs, regulatory T cells; UC, ulcerative colitis.
