# Supplementary material for: Mesalamine-Mediated Amelioration of Experimental Colitis in Piglets Involves Gut Microbiota Modulation and Intestinal Immune Cell Infiltration
Source: Front Immunol. 2022 Jul 8;13:883682. doi: 10.3389/fimmu.2022.883682 (PMC9309220; doi:10.3389/fimmu.2022.883682)
Supplement: Supplementary file 1 [file DataSheet_1.docx]

Supplementary Material

# Supplementary Figures and Tables

## Supplementary Figures


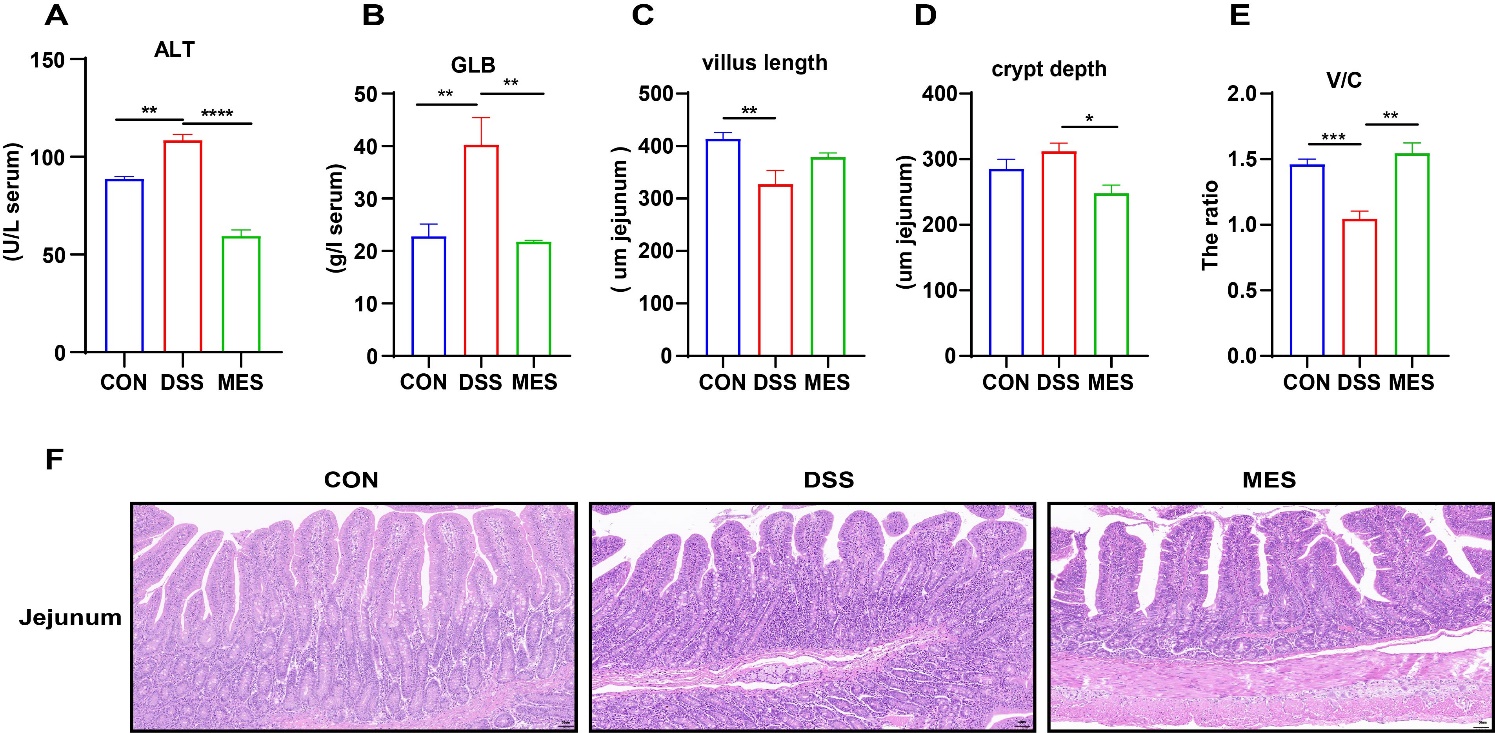


**Supplementary Figure 1.** MES affects the jejunal morphological changes in piglets with DSS-induced colitis. Serum levels of **(A)** ALT and **(B)** GLB. **(C)** jejunal villus length, **(D)** crypt depth, and **(E)** the ratio of villus length to crypt depth, V/C. **(F)** H&E-stained jejunum sections. Scale bar = 50 µm. Data were presented as mean ± SEM (n = 6 per group). Statistical significance was determined using one-way ANOVA, followed by Tukey test. **P* < 0.05, ***P* < 0.01, ****P* < 0.001, *****P* < 0.0001.


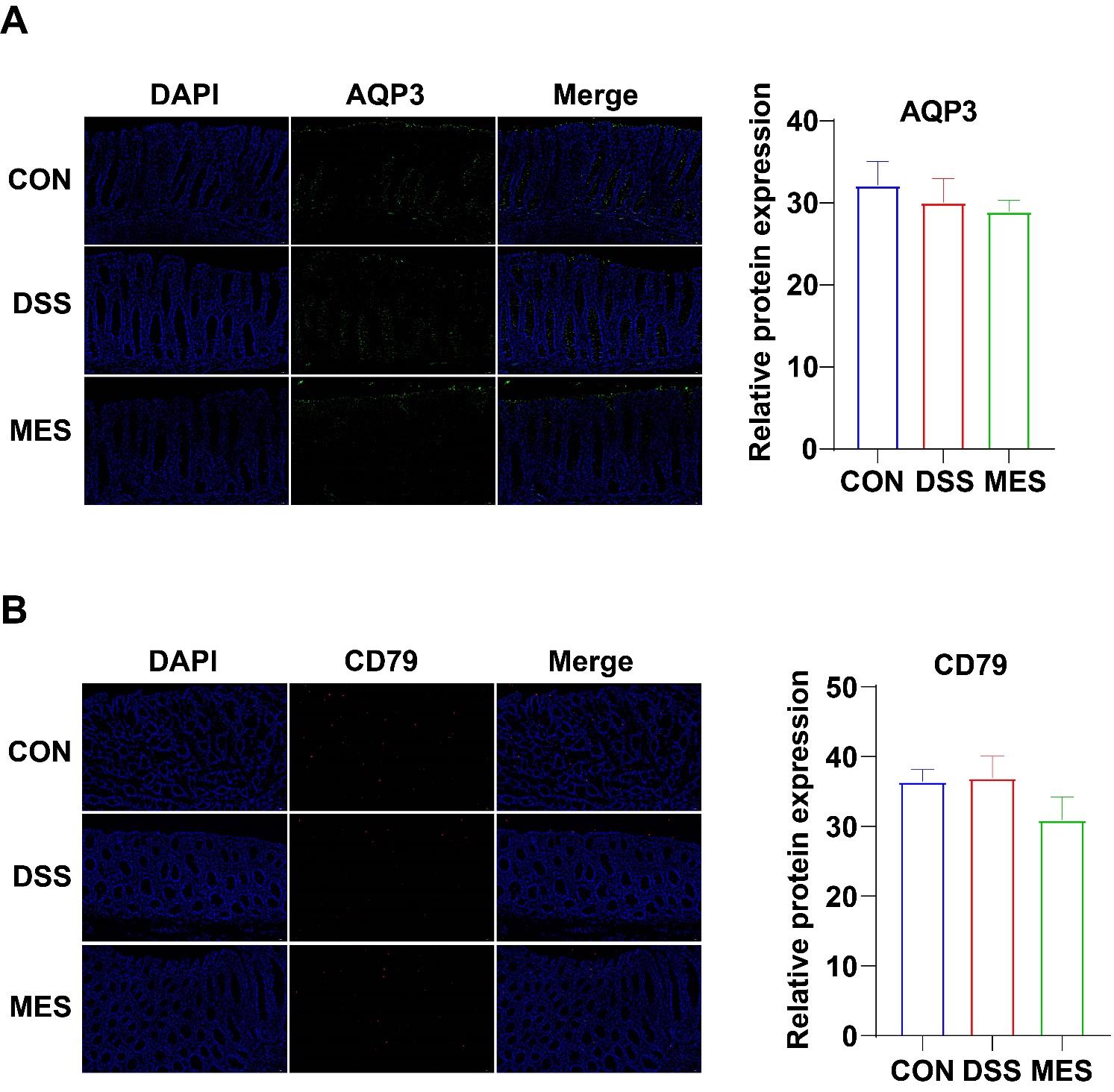


**Supplementary Figure 2.** MES treatment has no effect on colonic CD79a^+^ B cells infiltration and AQP3 expression. Relative protein expressions of **(A)** AQP3 and **(B)** CD79a, and representative images are shown. Scale bar = 50 µm. Data were presented as mean ± SEM (n = 6 per group). Statistical significance was determined using one-way ANOVA, followed by Tukey test.


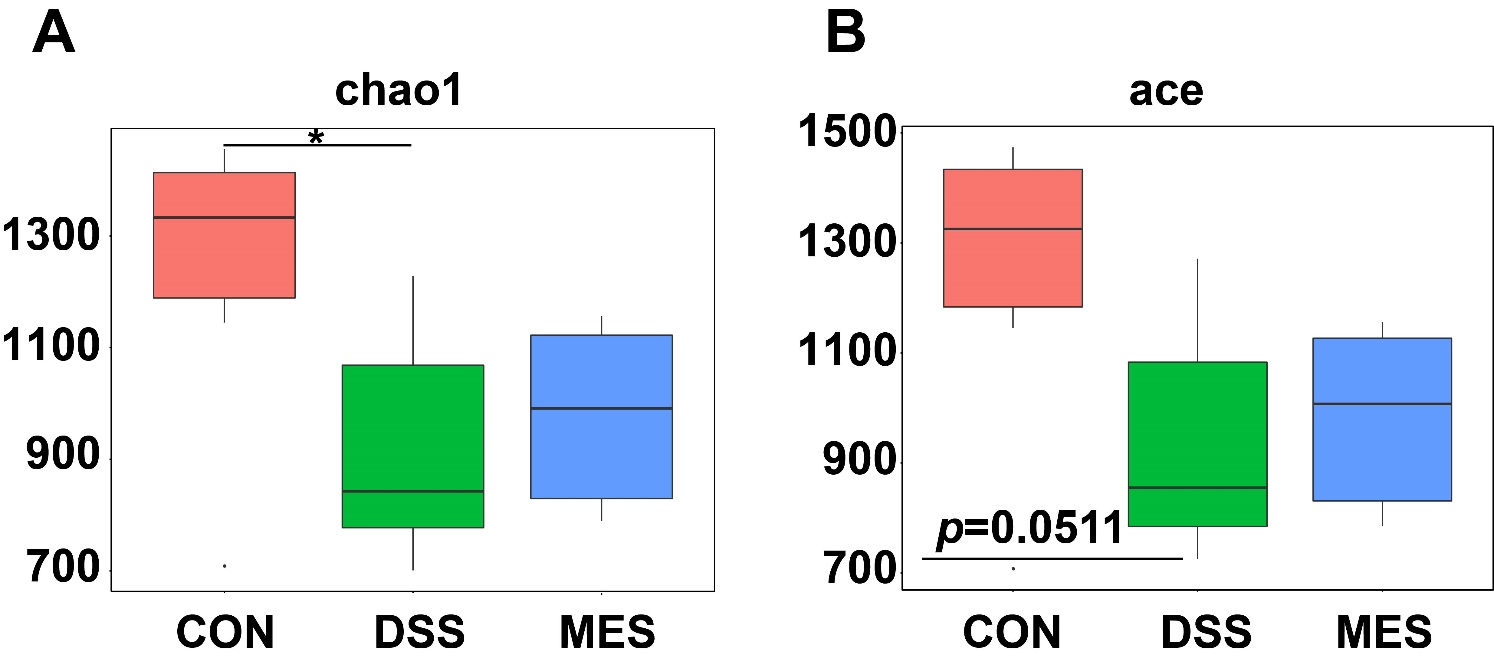


**Supplementary Figure 3.** Alpha diversity index. **(A)** chao1 and **(B)** ace. **P* < 0.05.

## Supplementary Table 1

Primers used in this study.

| **Gene Name** | **Accession No.** | **Sequence (5'-3')** | **Size (bp)** |
| --- | --- | --- | --- |
| IFN-γ | [NM_213948.1](https://www.ncbi.nlm.nih.gov/entrez/viewer.fcgi?db=nucleotide&id=47522725) | F: GAAGAATTGGAAAGAGGAGAGTGA  R: GCTCCTTTGAATGGCCTGGT | 109 |
| IL-6 | [NM_214399.1](https://www.ncbi.nlm.nih.gov/entrez/viewer.fcgi?db=nucleotide&id=47523851) | F: CCCTGAGGCAAAAGGGAAAGA  R: CAGGTGCCCCAGCTACATTA | 194 |
| IL-17A | [NM_001005729.1](https://www.ncbi.nlm.nih.gov/entrez/viewer.fcgi?db=nucleotide&id=54020963) | F: GAGATCCTCGTCCCTGTCAC  R: TGGAGAGTCCATGGTGAGGT | 213 |
| IL-22 | [XM_021091968.1](https://www.ncbi.nlm.nih.gov/entrez/viewer.fcgi?db=nucleotide&id=1191879964) | F: TCACCGAAACAGGTTCTCCT  R: CTCCAGAAGGAAAGGGCTCC | 100 |
| IL-1α | NM_214029.1 | F: AACCTGGATGAGGCAGTGAA  R: AACACGGGTTCGTCTTCGTT | 131 |
| IL-1β | NM_001302388.2 | F: AAAGGCTCCAACTTCCCCAGA  R: GAGTGTAGGTCCTGCCACAAA | 98 |
| CXCL11 | NM_001128491.1 | F: GCTACAACTATTCAAGGCTTCCC  R: TGCTTTTACTCCAGGGCCAAT | 72 |
| CXCL9 | NM_001114289.2 | F: TGATTGGAGTTCAAGGAACCCT  R: TCTCACAAGAAGGGCTTGGG | 129 |
| CCL21 | NM_001005151.1 | F: GCTATGTGCAGACCCCCAAA  R: GGGGGTCTCAGTCCTCTTACA | 166 |
| FOSB | XM_021094557.1 | F: CGAGAACGGAACAAACTGGC  R: AACTGGTCTGTCTCCGCCTG | 86 |
| TNFSF8 | NM_001025219.1 | F: GCCTACCTCCAAGTGTCAAAG  R: CCAACCAGGGAACTGGATCA | 117 |
| ACTB | [XM_003124280.5](https://www.ncbi.nlm.nih.gov/entrez/viewer.fcgi?db=nucleotide&id=1191864133) | F: CTGCGGCATCCACGAAACT  R: AGGGCCGTGATCTCCTTCTG | 147 |

Forward: F, Reverse: R.
